# Induction of heartwood formation in young Indian sandalwood (*Santalum album* L.) by gas elicitors

**DOI:** 10.3389/fpls.2022.961391

**Published:** 2022-07-28

**Authors:** Xiaojin Liu, Qilei Zhang, Zhou Hong, Daping Xu

**Affiliations:** Research Institute of Tropical Forestry, Chinese Academy of Forestry, Guangzhou, China

**Keywords:** carbon dioxide, ethylene, heartwood formation, gas elicitor, *Santalum album*

## Abstract

Induction of heartwood formation in 6-year-old Indian sandalwood (*Santalum album* L.) trees by treatment with carbon dioxide, ethylene, nitrogen, and wounding was investigated. All treatments induced fragrant heartwood formation upward and downward from the drill hole. The amount of heartwood formed above and below the drill hole depended on the treatment in the order nitrogen>carbon dioxide>ethylene>wounding, whereas the radial extension proportion was, in order, nitrogen>carbon dioxide>ethylene=wounding. Based on the chemical analysis (GC–MS) and evaluation of the essential oil quality and heartwood properties, heartwood induced by carbon dioxide showed the maximum similarities to naturally formed heartwood, which included the same color, similar chemical composition, reasonable oil content, and quality essential oil, whereas ethylene, nitrogen, and wounding treatment showed fewer similarities to natural heartwood. The results suggest that carbon dioxide is a promising candidate gas elicitor for inducing heartwood formation in young *S. album*.

## Introduction

In most hardwood tree species, the trunk wood can be divided into living sapwood and dead heartwood. Heartwood lies in the inner layers of the secondary xylem, has ceased to contain living cells, and is characterized by the accumulation of extractives (oils, phenols, gels, pigments, other heartwood substances, etc.). Sapwood contains living cells (ray parenchyma and axial parenchyma) and is characterized by a high content of reserve materials (Anon, 1957; IAWA, 1964; Galibina et al., 2020). These differences in composition result in the significant variations in color, density, durability, odor, moisture content, and permeability (Taylor et al., 2002; Tae et al., 2017). As a result, the applications as well as economic values of heartwood and sapwood differ markedly (Johannes and Peer, 2008; Miranda et al., 2017; Tae et al., 2017; Cui et al., 2020).

Sandal (*Santalum album* L.), commonly known as East Indian Sandalwood, is the most famous valuable tree species belonging to the Santalaceae family and is highly valued for its fragrant heartwood, which has various applications in the perfumery, religion, cosmetic, wood carving, and pharmacological industries (Nicolas et al., 2011; Misra and Dey, 2013; Haque and Coury, 2018; Mohankumar et al., 2019; Pullaiah et al., 2021). *S. album* was first introduced into China in 1962 (Li, 2003) and has exhibited encouraging growth performance in most planting areas in the recent decades in southern China (Liu et al., 2011, 2016). However, very few sandal plantations formed aromatic heartwood naturally when they were young (< 10 years old), and the quality of essential oil distilled from those young sandals was quite poor (Liu et al., 2011, 2012). Therefore, induction of fragrant heartwood formation in young sandalwood plantations has great importance in large-scale cultivation practices because the values of sandalwood at harvest will depend largely on the volume and the quality of heartwood. Several studies have shown that some chemical elicitors, such as CuSO_4_ (Kadambi, 1954), ethrel (Li and Chen, 1994), paraquat (Radomiljac, 1998), benzyladenine (Liu et al., 2013), and H_2_O_2_ (Li et al., 2021), can induce heartwood formation in young sandals. However, all these chemical elicitors were used as liquid solutions, and the induced heartwood was generally formed around the injection holes; moreover, the amount was quite low.

Stem gases play the important roles related to many physiological or metabolic processes in trees, such as aerobic respiration, the tricarboxylic acid cycle, secondary metabolism, and heartwood extractive accumulation, which can further regulate heartwood formation (Carrodus, 1967; Eklund, 2000; Spicer and Holbrook, 2007; Johannes and Peer, 2008). For instance, when branches from *Acacia mearnsii* containing only sapwood were exposed to a carbon dioxide (CO_2_) atmosphere, heartwood flavonoids specific for *A. mearnsii* were induced (Carrodus, 1971). Stem filling with ethylene (C_2_H_4_) can induce discolored heartwood formation in *Pinus sylvestris*, and the chemical composition of induced the heartwood was quite similar to that of naturally formed heartwood (Nilsson et al., 2002). Johannes and Peer (2008) noted that oxygen could cause red heartwood formation in European beech (*Fagus sylvatica*) by affecting the activities of microorganisms in standing stems and suggested that there is a threshold for oxygen concentration to trigger red heartwood formation.

Compared to liquid elicitors, gaseous elicitors seem to induce a larger amount of heartwood because pressurized gases can permeate almost everywhere in stems or branches (Nilsson et al., 2002). Therefore, it is necessary to test whether gas elicitors can induce fragrant heartwood formation in young sandals. The aim of this study was to determine whether and how gaseous elicitors (CO_2_, C_2_H_4_, and N_2_) influence fragrant heartwood formation in young sandal.

## Materials and methods

### Experimental site and tree selection

The field experiment was conducted in a sandalwood experimental and demonstration base of the Research Institute of Tropical Forestry, Chinese Academy of Forestry, which is located approximately 15 km north of Zhaoqing City, Guangdong Province, southern China (112°36′ E, 22°54′ N). The mean temperature is 23°C, and the annual rainfall is 1,700 mm. The plantation was first established in April 2004 in a laterite soil type with a sandalwood spacing of 3 × 3 m, and the main hosts were *Caesalpinia sappan* L. (Caesalpiniaceae), *Clausena lansium* (Lour.) Skeels (Rutaceae), *Ligustrum lucidum* Ait. (Oleaceae), and *Murraya exotica* L. (Rutaceae), one of which was planted between two sandalwoods with a spacing of 3 × 3 m. A preliminary study showed that this area was suitable for sandalwood growth and that the trees formed heartwood naturally at an early age (Liu et al., 2011).

A total of thirty sandals (*S. album*) without natural heartwood formed were selected randomly from a 6-year-old sandalwood plantation planted in the above sandalwood experimental and demonstration base. The presence of natural heartwood was determined by the color and odor of wood chips drilled through the central bottom of each sandal using a hand drill. The average height was 5.14 ± 0.43 m, and the average diameter on breast height (DBH) was 8.52 ± 0.41 cm. The selected trees were then divided into three gas treatment groups, one wounding group and one control group, and each treatment consisted of 6 individuals.

### Gas treatment

**Figure 1:**
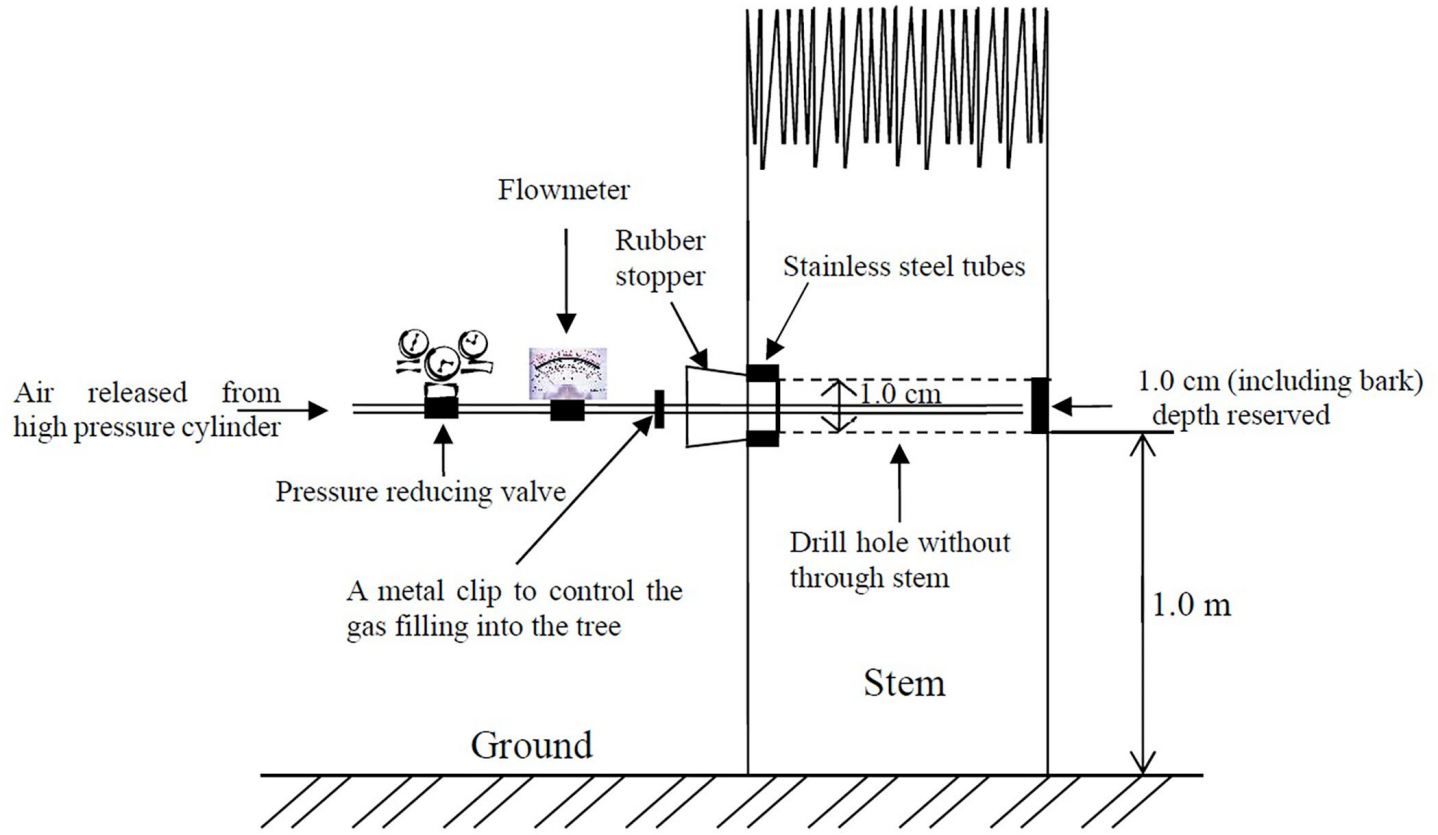
Schematic map for treatment of sandal with different gases.

Holes measuring 10 mm in diameter were drilled (without drilling through the trunk) using a hand drill at 1-m height above ground. The depth of each hole was calculated as the diameter minus 1 cm (Figure 1). After drilling, a rubber stopper equipped with a stainless steel tube was sealed on the entrance of each hole, so that a specific environment based on the gas treatment was created through the stem of each sandal, and thus, the volume of each hole was calculated. Drilling and tube insertion were conducted in April, so that the wounds had enough time to heal before treatment began in September, just before the onset of heartwood formation. Pressurized gases filled in different steel cylinders were released by a pressure reducing valve to maintain a steady flow (the air pressure was ~0.2 MPa), and a flowmeter equipped with a metal clip was placed between the valve and the rubber stopper to control the volume of gas filling the stem (Figure 1). Before filling, we opened the rubber stopper and released the corresponding gases from deep in each hole for 5 min to ensure that the residual gases were exhausted. Then, we closed the rubber stopper and sealed it with paraffin. A total of two volumes of gases (determined and controlled by a flowmeter and a digital timer) were filled into each hole and then stopped with a metal clip. Preliminary tests with ethylene showed that ethylene-filled trunks can still maintain a 15% concentration of the gas after 7 days, so this procedure was repeated one time a week for 1 year. A total of three types of gases were tested in this experiment: carbon dioxide (99.999%, CO_2_), ethylene (99.9%, C_2_H_4_), and nitrogen (99.999%, N_2_). Nitrogen was applied to create a hypoxic environment, wounding group consisted of sandals that remained untouched during the whole experiment after the holes drilled, and another control group kept the same (without drilling hole), just to observe whether heartwood formed naturally during the experiment.

### Sample collection and heartwood extension determination

An increment borer (Haglof, C300, 5.15 mm inner diameter, Sweden) was applied to draw two core samples from each tree at approximately 1 cm above the hole and at right angles to each other. The presence of heartwood and the length of heartwood and sapwood were determined and recorded. To determine the maximum extension distance of heartwood in each tree, we repeated the core sample collection procedure every 5 cm above or below the hole until no heartwood was observed. Using the above data, the whole extension area upward and downward from the hole can be simulated by drawing software (SigmaPlot, Version 13.0). All the cores were sealed separately with parafilm and then stored in a cooled insulated container before being transported to the laboratory, where the essential oil content and oil composition were determined.

### Heartwood property determination

The basic density and relative water content of the induced heartwood were determined immediately after core sample collection. The basic density (bone-dry weight per unit of fresh volume) was determined using the water displacement method, and the dry weight was obtained after drying at 105°C to constant weight. The water content (WC, express as percentage) was calculated as the percentage of water content per fresh weight (Searle and Owen, 2005).

### Essential oil extraction and content determination

Sapwood was removed from the collected cores using a knife, and the remaining induced fresh heartwood was weighed (FHW) and then ground into fine particles. Essential oil was extracted from the heartwood powder using the solvent (diethyl ether) extraction method (Howes et al., 2004). The diethyl ether solution was dried over anhydrous sodium sulfate and evaporated on a rotary evaporator under reduced pressure to give a yellow oil, and then, the weight of oils for each individual was measured separately (OW). Finally, the essential oil content was calculated by the dry weight (oil content of each dry heartwood, DW) and fresh weight (oil content of each fresh heartwood, FW) separately according to the following formula (Haffner, 1994):


$$\begin{array}{l} \text{Essential oil content }(\text{DW})\%\\ =\frac{Oil\;weight\;(OW)}{Fresh\;heartwood\;weight\;(FHW)\times (100-WC)}\times 100\\ \text{Essential oil content }(\text{FW})\%\\ =\frac{Oil\;weight\;(OW)}{Fresh\;Heartwood\;weight\;\left(FHW\right)}\times 100 \end{array}$$

where:


WC=water content of the induced heartwood.

### Essential oil composition determination

The essential oil composition extracted from the induced heartwood was determined by gas chromatography–mass spectrometry (GC–MS) according to the methodology by Howes et al. (2004) and Liu et al. (2011). The solvent (diethyl ether) was obtained from Aldrich, China. The GC–MS analysis was performed on a Finnigan TRACE GC-2000-MSTM (USA) instrument equipped with a DB-5 column (Agilent, USA, 30 m length, 0.25 mm inner diameter, and 0.25-μm film thickness). The injection port temperature was 220°C, and the oven initial temperature was 45°C. The oven temperature was ramped from 45°C at 3°C /min to 220°C and held for 5 min (total run time 63 min). The carrier gas was helium with a flow rate of 1.0 ml/min, and no split was used. All oils were diluted to 1.0% (v/v) with diethyl ether prior to analysis. The total injection volume was 1 μl. The mass spectra were fitted with an EI source operated at 70 eV with a source temperature of 200°C, and the mass spectra were recorded in the range m/z 35–335 amu at 1 scan/0.75 s. Each compound (peak) was identified either from retrieving the National Institute of Standards and Technology (NIST, Gaithersburg, MD, USA) standard mass spectra libraries (NIST2005, NIST2005s, NIST2014, and NIST2014s) or by comparing retention indices and/or mass spectra with published data (Verghese et al., 1990; Howes et al., 2004; Shellie et al., 2004; Jones et al., 2006; Sciarrone et al., 2011). The relative content of each compound was estimated according to the relative area through an area normalization method.

### Statistical analysis

Analysis of variance (ANOVA) was performed on each dataset using the statistical program SPSS 18.0 (SPSS I, 2004) after examining the data graphically to confirm normality of distribution and homogeneity of variances. Duncan's multiple range test was used to compare the differences between treatment means (α = 0.05).

## Results

### Heartwood formation and extension

**Figure 2:**
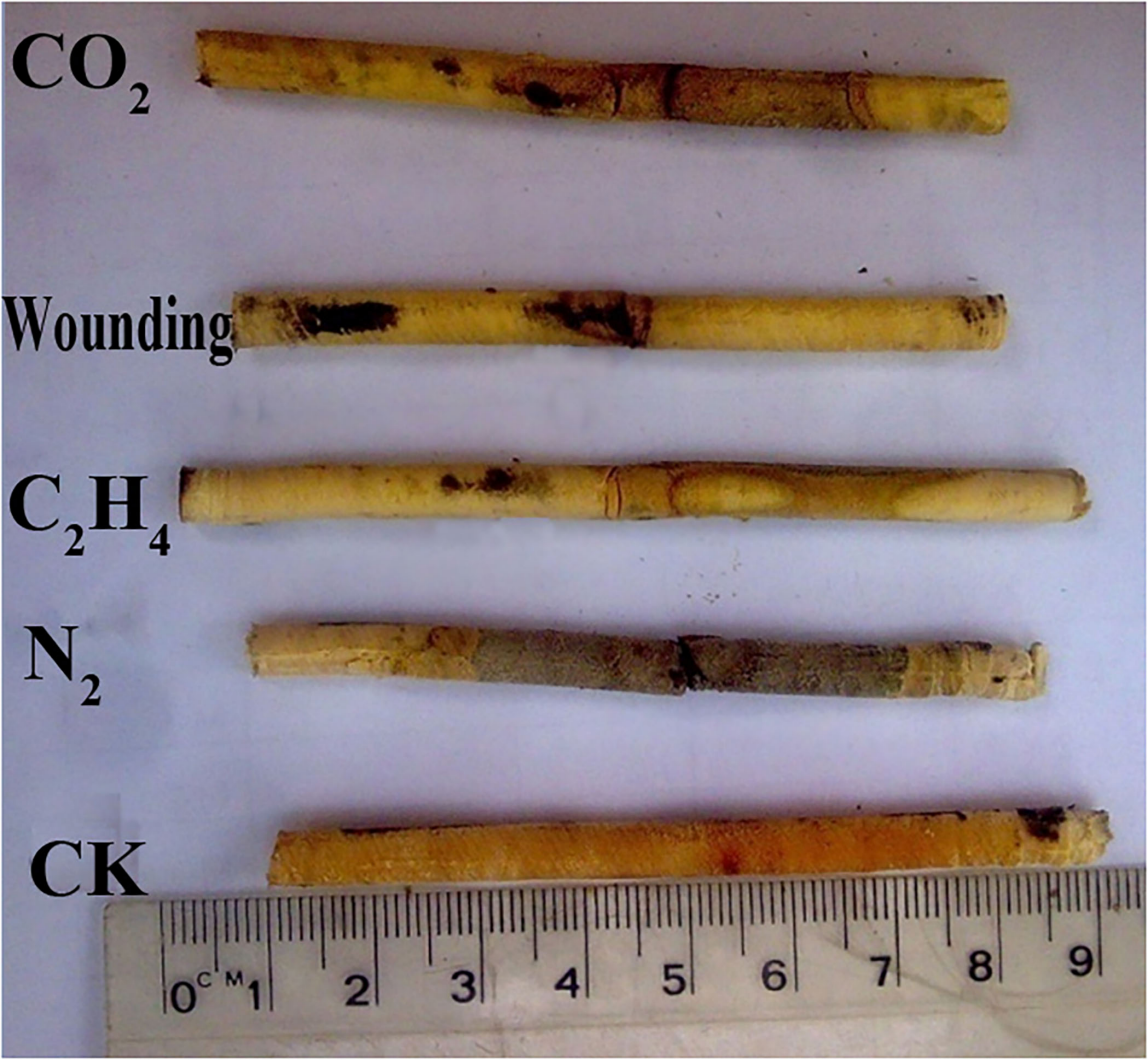
Core samples induced by different gas treatments (CO_2_, wounding, C_2_H_4_, and N_2_). Cores were taken from 1 cm above the drill hole; CK corresponds to the control group of young sandals at the same height. Ruler = 10 cm.

**Figure 3:**
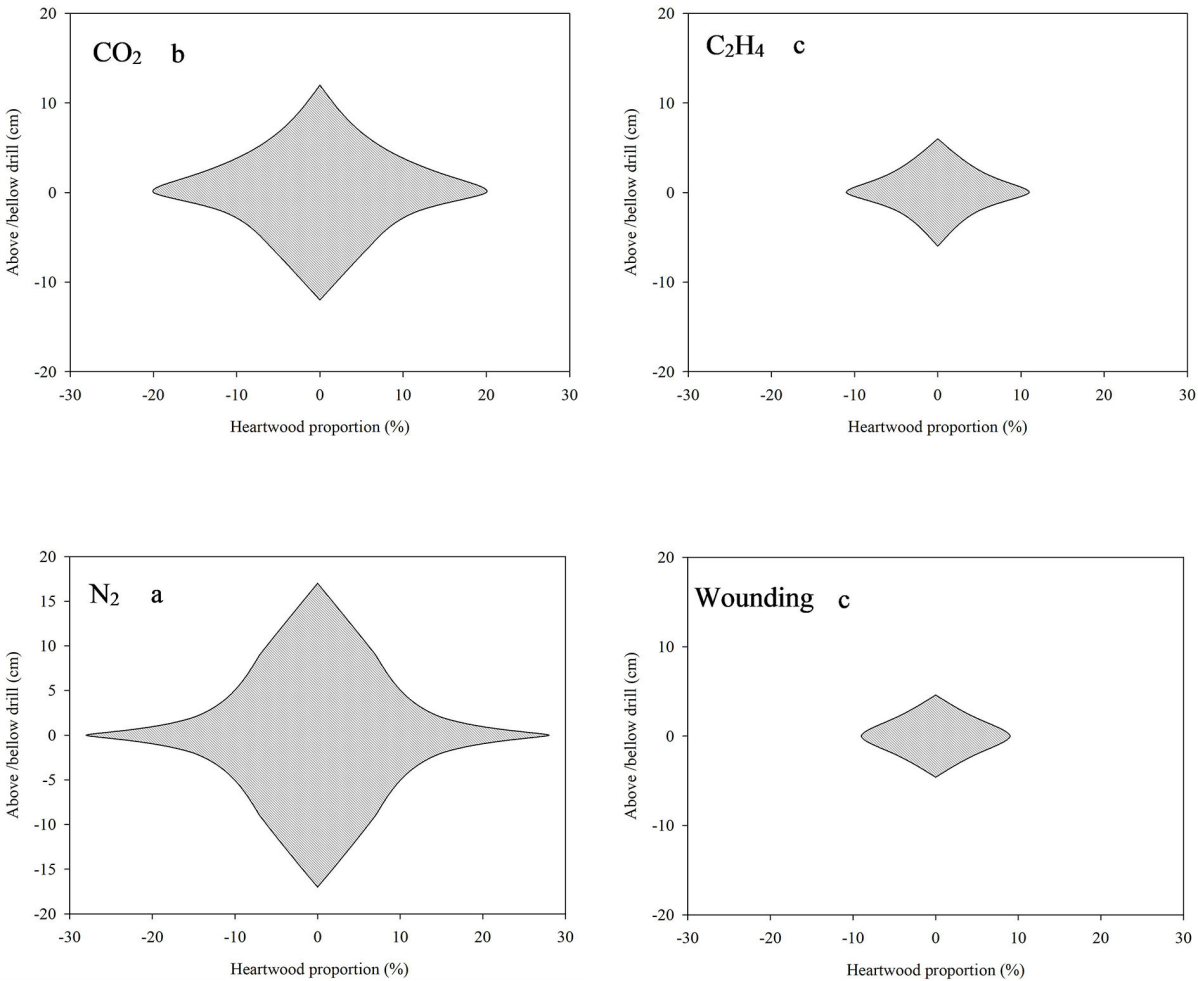
Longitudinal and radial extension of heartwood induced by different gas treatments. Longitudinal extension is expressed as the distance above/below the drill hole (cm), and radial extension proportion is expressed as the width of induced heartwood relative to the whole wood (%). Different letters indicate significant differences at *p* < 0.05.

All treated sandals formed fragrant heartwood 1 year after treatment (Figure 2), but the longitudinal extension range (estimated by the distance upward and downward from the drill hole), radial extension proportion (expressed as the maximum width of heartwood relative to the whole wood), and heartwood color differed greatly among treatments (Figures 2, 3). The greatest upward and downward extensions of induced heartwood from the drill holes were observed in trees treated with nitrogen (N_2_), which showed the significant differences when compared to the other treatments (Figure 3, *p* < 0.05), and the corresponding extension ranges were 16 and 17 cm, respectively. The maximum radial extension proportion of induced fragrant heartwood was 56% on average; however, the color of induced heartwood seems to be paler or lighter than that of naturally formed heartwood (Brand et al., 2012) (Figure 2). Carbon dioxide (CO_2_) induced less extended heartwood than N_2_, the corresponding upward and downward extension ranges were 11 and 12 cm, respectively, and the radial extension proportion of induced heartwood was 45% on average, but the color of induced heartwood was quite similar to that of naturally formed heartwood. The ethylene (C_2_H_4_) and wounding treatments induced the least amount heartwood, and the upward and downward extensions from the drill holes were quite similar (~7–8 and 4–5 cm, respectively). The radial extension proportions of C_2_H_4_-induced and wounding-induced heartwood were 24 and 22%, respectively. The amount of fragrant heartwood induced by C_2_H_4_ was slightly higher than that induced by wounding in both the longitudinal extension range and radial extension proportion; however, no significant difference was observed (Figure 3). The color of heartwood induced by C_2_H_4_ was slightly darker than that of naturally formed heartwood, but the wounding treatment exhibited quite similar color to the naturally formed heartwood of sandal (Figure 2). However, no heartwood was observed in all the control group at the same height (Figure 2, CK), which may indicate that 7-year-old sandalwood can hardly form heartwood naturally at the height of 1 m, and thus, the following indices such as heartwood properties, essential oil content, and oil composition cannot be determined and compared in this study.

### Heartwood properties

The results of the different gas treatments on the properties of induced heartwood are shown in Table 1. The ANOVA results showed that there was no significant difference among the gas treatments in either the water content (*p* = 0.747) or basic density (*p* = 0.996) of the induced heartwood in young sandals (α = 0.05). Treatment with C_2_H_4_ resulted in the highest water content and basic density (Table 1).

**Table 1:** Effects of different gas treatments on wood property of induced heartwood in young sandals^*^.

**Treatments**	**Water content (%)**	**Basic density (g.cm** ^−3^ **)**
CO_2_	27.40 ± 1.87a	0.587 ± 0.080b
C_2_H_4_	30.95 ± 4.35a	0.603 ± 0.027b
N_2_	28.04 ± 8.94a	0.595 ± 0.087b
Wounding	26.61 ± 6.03a	0.592 ± 0.146b

* *Data are shown as means ± standard deviation. Values with the same letters are not significantly different from each other according to Duncan's multiple comparison (α = 0.05)*.

### Essential oil content

**Figure 4:**
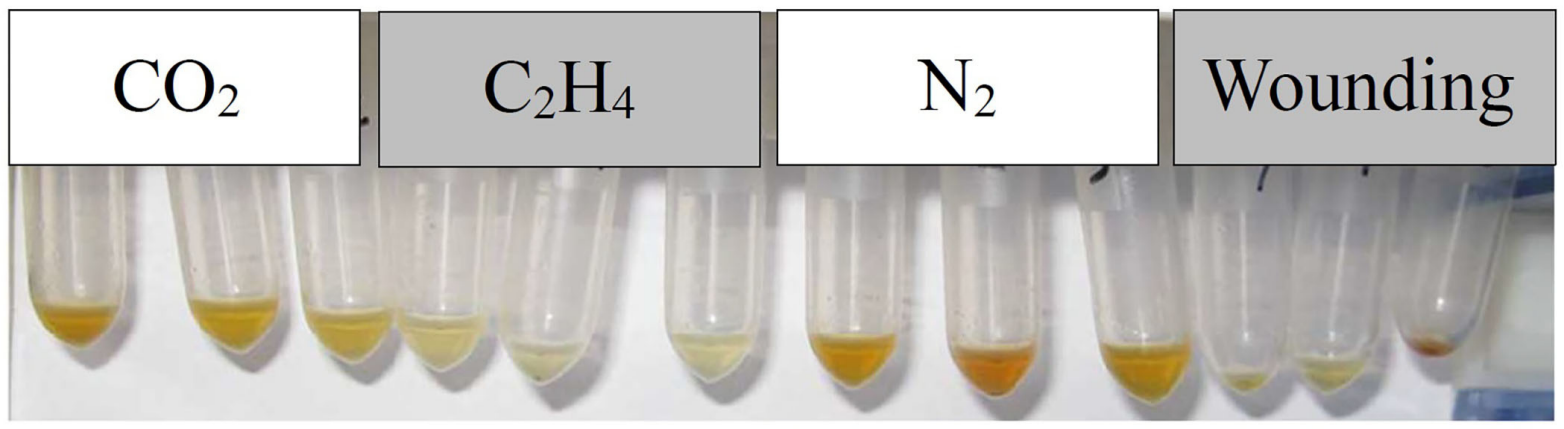
Essential oil extracted from heartwood induced by different gases in young sandals.

**Figure 5:**
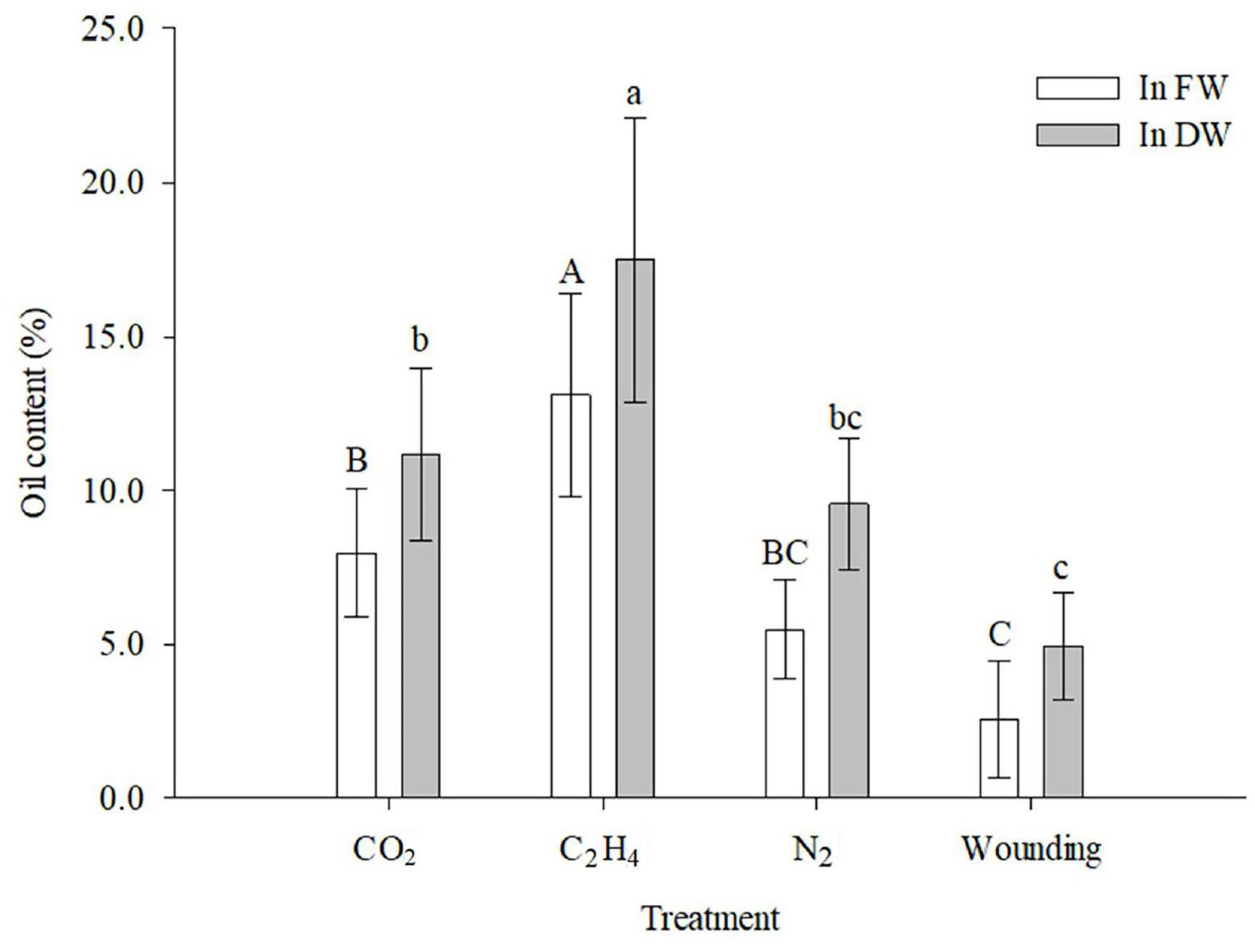
Effects of different gas treatments on essential oil content. Data are shown as means ± standard deviation. Values with the same superscript letters are not significantly different according to Duncan's multiple comparisons test (α= 0.05).

Great differences were observed in the essential oil content (*p* = 0.003) of the induced heartwood among the gas treatments according to the ANOVA, and the color of the extracted essential oil was different in shade (Figure 4). Treatment with C_2_H_4_ resulted in a statistically higher content than all the other treatments (Figure 5), and the corresponding essential oil contents were 13.11% in fresh weight and 17.25% in dry weight. The CO_2_ and N_2_ treatments induced less essential oil content than the treatment with C_2_H_4_, and the essential oil content did not differ significantly from each other. The wounding treatment resulted in the lowest essential oil content, only 2.26% in fresh weight and 5.02% in dry weight.

### Essential oil composition

**Figure 6:**
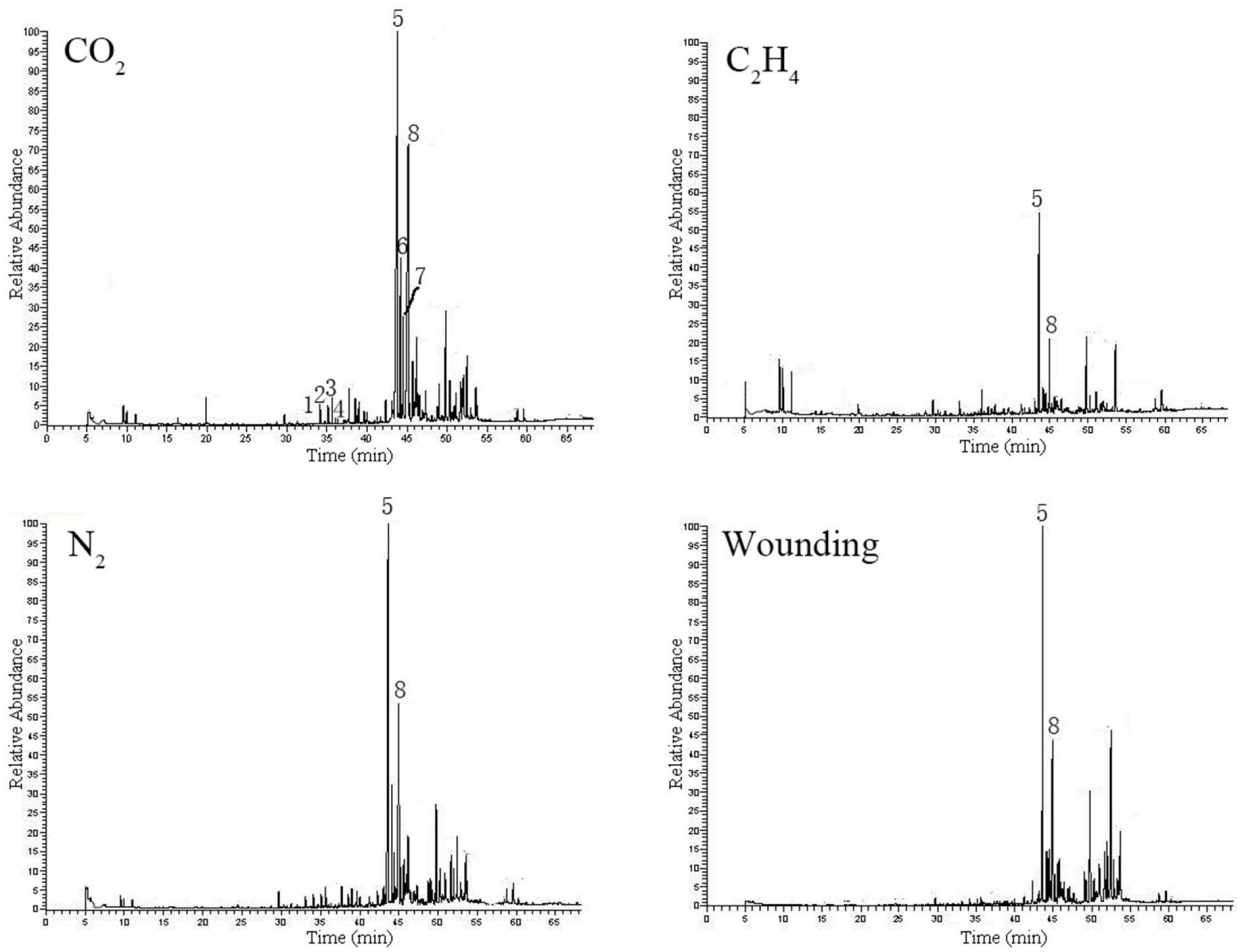
Total ion chromatogram of essential oil extracted from sandal induced by gas elicitors. 1: α-santalene 2: α-bergamotene 3: epi-*β*-santalene 4: *β*-santalene 5: α-santalol 6: α-*trans*-bergamotenol 7: epi-*β*-santalol 8: *β*-santalol.

The GC–MS analysis revealed that 38 peaks were detected in all the essential oils extracted from heartwood induced by the different gas treatments, of which 31 components were identified and confirmed. Details on the constituents identified from the essential oils treated with the different gases are listed in Table 2, and the total ion chromatogram of each treatment is shown in Figure 6. Significant differences (*p* < 0.01) were observed among the different gas treatments in the content of the main characteristic compounds of the essential oil *viz*. α-santalene, α-bergamotene, epi-*β*-santalene, *β*-santalene, α-santalol, and *β*-santalol according to the ANOVA (Table 2, Figure 6).

**Table 2:** Chemical composition of essential oil extracted from heartwood induced by differnet gas treatments.

**Retention time (min)**	**Compound name**	**Molecular formula**	**Relative content(%)**			
			**CO**_2_	**C**_2_ **H**_4_	**N**_2_	**Wounding**
9.45	Isopropyl acetate^*^	C_5_H_10_O_2_	1.83 ± 1.45b	4.56 ± 3.66a	1.14 ± 0.56b	–
9.96	3-Ethoxy-2-butanone^*^	C_6_H_12_O_2_	0.70 ± 0.34b	2.13 ± 1.75a	0.48 ± 0.23b	0.37 ± 0.45b
11.01	Acetal^*^	C_6_H_14_O_2_	0.71 ± 0.36b	2.04 ± 1.57a	0.47 ± 0.22b	0.32 ± 0.44b
19.06	Nononal	C_9_H_18_O	–	0.37 ± 0.36	0.13 ± 0.06	0.14 ± 0.07
19.75	Glycerol	C_3_H_8_O_3_	0.75 ± 0.71	0.83 ± 0.79	0.32 ± 0.16	–
24.00	Decanal	C_10_H_20_O	0.06 ± 0.01	0.14 ± 0.07	0.10 ± 0.06	0.17 ± 0.17
27.89	n-Docosane	C_22_H_46_	0.07 ± 0.04	0.31 ± 0.11	0.10 ± 0.07	0.25 ± 0.18
28.68	n-Eicosane	C_20_H_42_	0.19 ± 0.08	0.83 ± 0.31	0.27 ± 0.20	0.68 ± 0.47
30.29	α-Sinensal	C_15_H_22_O	–	0.62 ± 0.38	–	–
33.12	Dodecanal^*^	C_12_H_24_O	0.18 ± 0.12c	1.21 ± 0.52a	0.80 ± 0.59b	0.74 ± 0.42b
34.13	α-Santalene^*^	C_15_H_24_	0.89 ± 0.14ab	1.26 ± 0.36a	0.55 ± 0.19bc	0.42 ± 0.07c
34.77	α-Bergamotene^*^	C_15_H_24_	0.93 ± 0.12a	1.02 ± 0.18a	0.43 ± 0.11b	0.31 ± 0.04b
35.13	Epi-*β*-Santalene^*^	C_15_H_24_	0.83 ± 0.16a	1.17 ± 0.31a	0.44 ± 0.13b	0.39 ± 0.05b
35.64	*β*-Santalene^*^	C_15_H_24_	1.25 ± 0.24ab	1.80 ± 0.49a	0.72 ± 0.21bc	0.53 ± 0.04c
36.37	α-Curcumene	C_15_H_22_	–	0.47 ± 0.20	–	–
37.34	2,4-Di-tert-butylphenol^*^	C_14_H_22_O	0.29 ± 0.23b	1.41 ± 0.75a	–	1.17 ± 0.81a
38.98	Teresantalol^*^	C_10_H_16_O	1.05 ± 0.51a	1.18 ± 0.64a	0.91 ± 0.28a	0.43 ± 0.20b
39.66	Lauric acid hydride^*^	C_24_H_46_O_3_	0.75 ± 0.47b	1.47 ± 0.85a	0.78 ± 0.16b	0.88 ± 0.70b
40.00	Farnesol	C_15_H_26_O	0.54 ± 0.18	0.79 ± 0.37	0.57 ± 0.25	0.56 ± 0.15
43.64	α-Santalol^*^	C_15_H_24_O	43.09 ± 2.10a	31.05 ± 5.03b	37.56 ± 1.30a	38.09 ± 2.91a
44.13	α-*trans*-Bergamotenol	C_15_H_24_O	6.65 ± 0.91	5.91 ± 2.58	6.84 ± 0.71	5.25 ± 1.36
44.44	Epi-*β*-Santalol	C_15_H_24_O	3.92 ± 1.13	2.38 ± 0.25	3.81 ± 0.17	3.46 ± 0.14
44.97	*β*-Santalol^*^	C_15_H_24_O	18.91 ± 2.73a	12.67 ± 3.57b	16.88 ± 2.32ab	16.39 ± 0.20ab
45.19	Heptacosane	C_27_H_56_	–	1.25 ± 0.69	–	–
46.45	*cis*-Lanceol	C_15_H_24_O	–	1.48 ± 0.33	–	–
51.97	Oxabicyclo[4.1.0] heptane,2,2,6-trimethyl-1-(3-methyl-1,3-butadienyl)-5-methylene-	C_15_H_22_O	2.36 ± 0.29	1.60 ± 1.46	3.08 ± 1.16	2.77 ± 1.70
53.69	Hexadecanic acid	C_16_H_32_O_2_	2.48 ± 0.69	5.35 ± 3.83	3.58 ± 1.25	3.32 ± 0.86
56.60	Dioctyl phthalate	C_24_H_38_O_4_	–	–	–	0.25 ± 0.20
58.48	Linolelaidic acid-methyl ester	C_19_H_34_O_2_	0.32 ± 0.31	–	0.28 ± 0.15	–
58.86	*trans*-9-Octadecenoic acid	C_18_H_34_O_2_	0.64 ± 0.39	1.34 ± 0.67	0.91 ± 0.24	–
59.68	Octadecanoic acid	C_18_H_36_O_2_	0.81 ± 0.18	2.75 ± 1.09	1.47 ± 0.49	1.02 ± 0.38
Total			90.23	89.38	82.27	77.91

Data are shown as means ± standard deviation. “–” means trace amount or not detected. Constitutes with “^*^” mean that there exists statistically different among all the gas treatment, and values with the same letters are not significantly different from each other according to Duncan's multiple comparison (α = 0.05).

Treatment with C_2_H_4_ obtained the highest number of identified ingredients (29 constituents) from the essential oil, followed by treatment with CO_2_ and N_2_, both of which had 25 identified constituents. The wounding treatment obtained the lowest number of identified ingredients with only 23 components identified. Although great variations in chemical composition and relative content were observed among the different treatments, the most dominant compounds in all gas treatments were found nearly in the same order: α-santalol > *β*-santalol > α-*trans*-bergamotenol > epi-*β*-santalol (Table 2, Figure 5).

The quality of the sandalwood essential oil was characterized by the presence of a high content of sesquiterpene alcohols and the corresponding sesquiterpenes. The ANOVA results showed that there were significant differences in the contents of total santalols (*p* = 0.015), total santalenes (*p* = 0.003), total sesquiterpenes (*p* = 0.039), and total sesquiterpene alcohols (*p* = 0.002). Treatment with CO_2_ was most favorable for santalol and sesquiterpene alcohol synthesis, whereas treatment with C_2_H_4_ was helpful for santalene and sesquiterpene accumulation (Table 3).

**Table 3:** ANOVA results of major ingredient group of essential oil induced by different gas treatments.

**Treatment**	**Relative content (%)** ^*^			
	**Total santalols**	**Total santalenes**	**Total sesquiterpenes**	**Total sesquiterpene alcohols**
CO_2_	65.92 ± 5.25 a	2.97 ± 0.51 b	3.90 ± 0.51 b	72.56 ± 6.13 a
C_2_H_4_	46.10 ± 6.83 c	4.23 ± 1.15 a	5.25 ± 1.08 a	53.49 ± 8.54 c
N_2_	58.25 ± 3.56 b	1.71 ± 0.53 c	2.15 ± 0.53 c	65.09 ± 3.89 b
Wounding	57.94 ± 3.37 b	1.35 ± 0.16 c	1.66 ± 0.18 c	63.19 ± 2.62 b

* *Data are shown as means ± standard deviation. Values with the same superscript letters are not significantly different from each other according to Duncan's multiple comparison (α = 0.05)*.

### Essential oil quality

The current International Organization for Standardization standard for *S. album* oil (ISO 3518:2002) requires that the essential oil contains both 41–55% α-santalol and 16–24% *β*-santalol (ISO, 2002), whereas the recommended quality for sandalwood oil to traded is α-santalol ≥43% and *β*-santalol ≥18% (Howes et al., 2004). Based on the above quality standard, only the treatment with carbon dioxide met the ISO standard for *S. album* oil and can also be traded on the international market. The contents of α-santalol and *β*-santalol were 43.09 and 18.91% on average, respectively (Table 2). This may also mean that stem filling with CO_2_ can induce or accelerate young sandals to form quality *S. album* oils, whereas the *S. album* oil produced by the other treatments (N_2_, C_2_H_4_, and wounding) did not meet the current ISO standards and cannot be traded on the international market.

## Discussion

Heartwood formation is a complicated biochemical process during tree growth and development, which includes the death of parenchyma, gas accumulation, lack of water transport, desiccation, embolism, pit closure, ethylene production, nutrient recycling, etc. (Taylor et al., 2002), and many hypotheses or theories, such as the pipe model (Shinozaki et al., 1964), homeostasis (Gartner, 1991), heartwood-inducing substances (Bamber, 1976; Cui et al., 2020), phytohormone stress (Nilsson et al., 2002), metabolism (Cui et al., 2021, 2022), pit membrane degradation (Crombie et al., 1985), and microbial infection (Johannes and Peer, 2008), have been proposed to interpret the mechanism of heartwood formation. However, it seems that the heartwood formation mechanism varies greatly by species (Bieniasz and Tulik, 2020), and too many factors can affect heartwood formation. In this study, the holes drilled in the trunk interrupted part of the xylem vessels, and water transportation driven by transpiration from the roots to the canopy was then affected. Consequently, the water potential in peripheral vessels was increased, and the filling with pressurized gases then penetrated the water-filled vessels through peripheral inter-vessel pits. As a result, cavitation or air embolism was occasionally formed in xylem vessels. With the continuous filling of pressurized gases, cavitation or air embolism occurred repeatedly, the pit membranes were damaged and subsequently degraded, and the heartwood (dead sapwood) was finally formed. Therefore, all the gas treatments (including wounding) in this experiment formed colored heartwood (Figure 2), and similar results were also found in *Populus tremuloides* (Sperry et al., 1991).

The heartwood extension area differed greatly among the gas treatments (Figure 3), which may be due to the different functions of these gases in live woody tissues. As an inert gas, nitrogen is rarely involved in primary and secondary metabolic reactions; it just entered the water-filled vessels and penetrated into the surrounding tissue through the frequent closure of pits, and finally, a large amount of pit membrane was damaged and degraded, which resulted in a larger area of induced heartwood. In the case of carbon dioxide and ethylene, both were involved in metabolic reactions related to heartwood formation. For instance, carbon dioxide can induce heartwood formation in *Acacia mearnsii* by synthesizing flavonoids (Carrodus, 1971), and ethylene has been widely applied to accelerate heartwood formation in many species, such as *Azadirachta indica* (Shah et al., 1981)*, Dalbergia odorifera* (Cui et al., 2019), and *Pinus sylvestris* (Nilsson et al., 2002). Under such situations, gases filling the trunk were partly depleted, and thus, the area of induced heartwood was less than that under nitrogen treatment. The reason that ethylene treatment induced less heartwood than carbon dioxide may be attributed to the greater depletion of ethylene in the heartwood formation of young sandal than carbon dioxide. Due to the lack of continuous filling of pressurized gases, cavitation or embolism rarely occurs, so the wounding treatment induced the least amount of heartwood (Figure 3); a similar phenomenon was also observed in *P. sylvestris* (Nilsson et al., 2002).

It is well-known that ethylene can increase the activities of phenylalanine ammonia-lyase, peroxidase, and terpene synthase, which are the important enzymes for polyphenol and terpene biosynthesis, and discolored heartwood is ultimately induced (Nilsson et al., 2002). In this experiment, significantly higher contents of phenols (Table 2), santalenes (Table 3), and sesquiterpenes (Table 3) were observed in the essential oil extracted from the induced heartwood treated with ethylene, which may indicate that ethylene accelerates the formation of sandal heartwood by stimulating the activities of phenylalanine ammonia-lyase, peroxidase, and terpene synthase. In the case of carbon dioxide, the accumulation of santalols (Table 2) and sesquiterpene alcohols (Table 3) was significantly stimulated, which may be explained by the consensus that high concentrations of carbon dioxide were helpful for the synthesis of ring structures in flavonoids and stilbenes (Carrodus, 1971; Higuchi, 1997). Although nitrogen does not participate in the metabolism of sandal heartwood formation, it causes hypoxia and thus may alter metabolism toward less oxygen-demanding reactions, which can also induce heartwood formation. Wounding treatment induced a small amount of heartwood, which may be attributed to the release of the stress ethylene (Nilsson et al., 2002; Liu et al., 2013). However, further study is needed to explore or confirm this hypothesis.

Oil yields of sandal heartwood are typically 3–8% through hydro-distillation (Jones and Plummer, 2007), whereas in the ethylene treatment, the essential oil content extracted from the induced heartwood was 13.11% in average (Figure 3), which was far higher than that from naturally formed heartwood. The extraction methods used in this study may be one of the reasons. Generally, solvent extraction method yielded more oil than the traditional hydro-distillation (Zhang et al., 2012; Liu et al., 2017), and this may due to the solvent can dissolve more substances than hydro-distillation. In this study, ethylene treatment got the highest oil content than all the other treatment, which may indicate that several ingredients with high boiling point were produced during heartwood formation, and these constitutes formed in the induced heartwood were extracted by the solvent (diethyl ether). As a result, higher content of essential oil was calculated and then observed. However, these ingredients cannot be isolated or detected through gas chromatography (GC), so we cannot find these substances in the total ion chromatogram (Table 2, Figure 6). Another may be related to the particular heartwood formation model of sandal (Type-III, Santalum) (Celedon and Bohlmann, 2017), which was quite different from Type-I (*Robinia*-type) and Type II (*Juglans*-type). The phenols and terpenes stimulated by ethylene may partly accumulate in the newly formed heartwood (Celedon et al., 2016) and then be extracted into the essential oils; therefore, a higher oil content was detected. Further studies focus on this issue were strongly demanded.

Although great differences were found in the above indices among the different treatments, the properties of induced heartwood were only slightly different (Table 1), which may indicate that the properties of sandal were relatively stable and less affected by external elicitors. Similar results were also observed in *D. odorifera* when induced by different plant growth regulators (Cui et al., 2021).

Based on the above quantitative and qualitative data, we suggest that carbon dioxide is an effective gas elicitor in the induction of heartwood formation in young sandal, and ethylene, nitrogen, and wounding are the potential regulators of heartwood formation in sandal, whereas in the case of *P. sylvestris* and *Fagus sylvatica*, ethylene and oxygen are the most important regulators (Nilsson et al., 2002; Johannes and Peer, 2008). However, the mechanism may differ greatly, and the mechanism underlying this regulation has not been investigated well. Thus, studies focusing on the metabolism and molecular mechanism of induction of heartwood formation are greatly necessary, as there are still too many unknown events or processes that occur during heartwood formation in trees.

## Conclusion

This study clearly demonstrated that gas elicitors played the important roles in controlling heartwood formation of young sandal. Stem filling with carbon dioxide can induce young sandal to form quality fragrant heartwood, which is consistent with naturally formed heartwood in color, properties, chemical composition, and essential oil quality. There may exist a link between carbon dioxide and the biosynthesis of santalol or sesquiterpenoids. Carbon dioxide is a promising candidate gas elicitor for inducing heartwood formation in young *S. album*.

## Data availability statement

The original contributions presented in the study are included in the article/supplementary material, further inquiries can be directed to the corresponding author.

## Author contributions

XL and DX conceived the ideas and designed the methodology. QZ collected the data. ZH analyzed the data. XL wrote the manuscript. DX supervised the project. All authors contributed critically to the drafts and gave final approval for publication.

## Funding

This research was sponsored by the Fundamental Research Funds for the Central Non-profit Research Institution of Chinese Academy of Forestry (CAFYBB2019QB003 and CAFYBB2016QB010) and the National Natural Science Foundation of China (Grant No. 31500512).

## Conflict of interest

The authors declare that the research was conducted in the absence of any commercial or financial relationships that could be construed as a potential conflict of interest.

## Publisher's note

All claims expressed in this article are solely those of the authors and do not necessarily represent those of their affiliated organizations, or those of the publisher, the editors and the reviewers. Any product that may be evaluated in this article, or claim that may be made by its manufacturer, is not guaranteed or endorsed by the publisher.
